# PMMA-TiO_2_ Fibers for the Photocatalytic Degradation of Water Pollutants

**DOI:** 10.3390/nano10071279

**Published:** 2020-06-30

**Authors:** Namrata Kanth, Weiheng Xu, Umesh Prasad, Dharneedar Ravichandran, Arunachala Mada Kannan, Kenan Song

**Affiliations:** 1 Materials Science & Engineering, School for Engineering of Matter, Transport and Energy (SEMTE), Ira A. Fulton Schools of Engineering, Arizona State University, Tempe, AZ 85281, USA; nkanth@asu.edu; 2 System Engineering, The Polytechnic School (TPS), Ira A. Fulton Schools of Engineering, Arizona State University, Mesa, AZ 85212, USA; weihengx@asu.edu (W.X.); uprasad1@asu.edu (U.P.); dravich2@asu.edu (D.R.); 3 The Polytechnic School, School for Engineering of Matter, Transport, and Energy (SEMTE), Ira A. Fulton Schools of Engineering, Arizona State University, Mesa, AZ 85212, USA; amk@asu.edu

**Keywords:** nanocomposite, fibers, TiO_2_, PMMA, photocatalytic

## Abstract

Titanium dioxide (TiO_2_) is a promising photocatalyst that possesses a redox potential suitable for environmental remediation applications. A low photocatalytic yield and high cost have thus far limited the commercial adoption of TiO_2_-based fixed-bed reactors. One solution is to engineer the physical geometry or chemical composition of the substrate to overcome these limitations. In this work, porous polymethyl methacrylate (PMMA) substrates with immobilized TiO_2_ nanoparticles in fiber forms were fabricated and analyzed to demonstrate the influence of contaminant transport and light accessibility on the overall photocatalytic performance. The influences of (i) fiber porosity and (ii) fiber architecture on the overall photocatalytic performance were investigated. The porous structure was fabricated using wet phase inversion. The core-shell-structured fibers exhibited much higher mechanical properties than the porous fibers (7.52 GPa vs. non-testability) and maintained the same degradation rates as porous structures (0.059 vs. 0.053/min) in removing methylene blue with comparable specific surface areas. The highest methylene blue (MB) degradation rate (*k*_MB_) of 0.116 min^−1^ was observed due to increases of the exposed surface area, pointing to more efficient photocatalysis by optimizing core-shell dimensions. This research provides an easy-to-manufacture and cost-efficient method for producing PMMA/TiO_2_ core-shell fibers with a broad application in water treatment, air purification, and volatile sensors.

## 1. Introduction

Ever-increasing pollution calls for the development of a robust and cheap solution for environmental remediation [1]. Among the numerous methods that can be employed to tackle this problem [2], harnessing the photocatalytic activity of titanium dioxide (TiO_2_) can offer a promising solution. TiO_2_ is a wideband semiconductor material that possesses a redox potential capable of decomposing organic contaminants into carbon dioxide and water [3]. This advanced oxidation capability not only makes it suitable for purification applications, such as pollutant capture [4], the treatment of industrial waste [5], and wastewater disinfection [6], but means that it can also be used for the reformation of hydrocarbon fuels [7]. The commercial adaptation of TiO_2_ has thus far been limited by a low throughput and the high cost of photoreactors [8,9]. These limitations can be addressed by a fixed-bed reactor where nano-scale TiO_2_ is immobilized on inert substrates. Nanoparticles of TiO_2_ provide a high surface to volume ratio, improving the transport of contaminants. Additionally, immobilization eliminates the need for expensive post-treatment separation of TiO_2_ [10]. However, this system suffers from drawbacks, such as a poor light distribution and hindered charge transfer, due to the encapsulation of TiO_2_ in substrates [11]. A cost-effective solution is to use polymers as a substrate, since they can be easily molded to the desired shape, size, and microstructure [12]. Past research has reported anchoring TiO_2_ on polymeric beads [11], membranes [13], and electrospun fibers [14]. Incorporating porosity in these substrates enhances mass transport. Another approach is to coat radially emitting optical fibers with TiO_2_ [15,16]. This unique architecture serves two purposes: It creates a greater surface area by utilizing the fiber’s longitudinal morphology and enables remote light accessibility through optical fibers [17]. However, to the best of our knowledge, previous studies have not compared the photocatalytic merits of improving mass transfer and enhancing light accessibility, which is discussed here.

**Figure 1:**
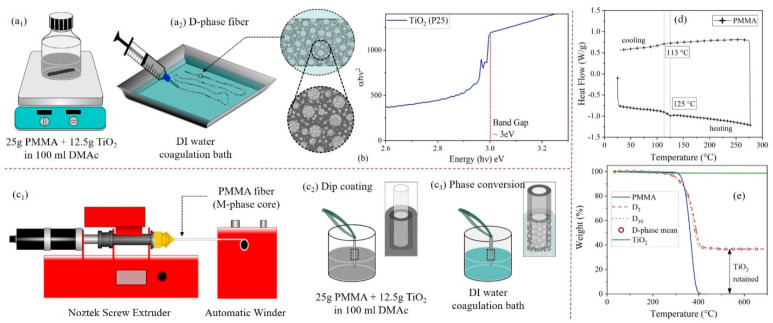
Schematic of the fabrication procedures for (**a_1_**,**a_2_**) preparing D-phase containing the (**b**) TiO_2_ with a bandgap of 3 ev (Tauc plot) and (**c_1_**) PMMA fiber extrusion, (**c_2_**) dip coating of extruded PMMA fiber and (**c_3_**) phase conversion for M-phase fibers. (**d**) Differential scanning calorimetry (DSC) of polymethyl methacrylate (PMMA) raw material. (**e**) Back-to-back thermogravimetric analysis (TGA) conducted to quantify TiO_2_ retention after wet phase inversion.

In this paper, we report the fabrication and characterization of polymethyl methacrylate (PMMA)-TiO_2_ fibers, manufactured by low-cost and scalable methods (Figure 1a–c). In this investigation, two fiber architectures were studied [18]. The first was a porous PMMA fiber containing dispersed TiO_2_ (D-phase), where the porous morphology entailed enhanced mass transport (Figure 1c_1,2_). The second was a multiphase fiber (M-phase) with a solid PMMA core and a porous PMMA-TiO_2_ sheath (Figure 1c_3_). The solid PMMA core in the M-phase serves as an optical fiber for remote light transmission, and the porous sheath provides enhanced mass transport similar to the D-phase. PMMA was chosen as a substrate material because of its excellent ultraviolet (UV) transparency [19] and stability against strong oxidative radicals [20]. Supporting materials, such as glass fibers, have been traditionally used for immobilizing TiO_2_; however, the rigidity and labor-intensive manufacturing of glass fibers mean that they are not optimal materials in water treatment applications [9,12,21,22,23]. The solid PMMA core was fabricated using melt spinning and the porous matrix (D-phase and M-phase sheath) was fabricated by wet phase inversion. Their photocatalytic performance was assessed by comparing the methylene blue (MB) concentrations in the dark and in the presence of UV (~365 nm) radiation. Inferences were drawn related to the effect of architecture/morphology on light transport and mass transport, which are critical parameters in reactor scale-up [9].

## 2. Materials and Methods

### 2.1. Chemicals

PMMA (M_W_~120,000), *N,N*-dimethylacetamide (DMAc, ACS Reagent 99%), tetrahydrofuran (THF) and MB (≥82% purity) were purchased from Sigma Aldrich (St. Louis, MO, USA). TiO_2_ (AEROXIDE^®^ TiO_2_ P 25, ~53 m^2^/g) was obtained from Evonik Industries (Essen, Germany). All polymers and solvents were used as received. Deionized (DI) water from a Millipore water purification system (Milli-Q Academic) (Sigma Aldrich, St. Louis, MO, USA) was used.

### 2.2. Characterization

The morphology of the fibers was observed using a Scanning Electron Microscope (SEM) (XL30 SEM-FEG) (Philips, Amsterdam, The Netherlands). For SEM, all samples were sputter-coated with 15 nm thick gold. The dispersion of TiO_2_ across the cross-section of the fibers was observed using Electron Dispersive Spectroscopy (EDS) (XL30 SEM-FEG) (Philips, Amsterdam, The Netherlands). The specific surface area (SSA) values were measured and obtained using Brunauer, Emmett, and Teller (BET, Tristar II 3020)(Micromeritics, Norcross, GA, USA) Surface Area and Porosity Analysis. The optical properties were measured using UV-vis spectrophotometry (UV-vis, GENESYS 150) (Thermo Fisher Scientific, Walham, MA, USA). Thermogravimetric (TGA) studies were conducted using TGA550 (TA instruments, New Castle, DE, USA) in an air atmosphere at a ramping rate of 20 °C/min from room temperature (RT) to 700 °C. Differential Scanning Calorimetry (DSC) was conducted in a nitrogen environment with a purging rate of 50 mL/min using DSC250 (TA instruments, New Castle, DE, USA). The mechanical property was characterized by conducting a tensile test using Discovery Hybrid Rheometer (HR-2) (TA instruments, New Castle, DE, USA) for ten fiber samples. For assessing the photocatalytic performance, MB concentration was monitored in the dark and in the presence of UV radiation. MB concentration was measured as per Beer-Lambert Law, by monitoring the absorption peak at 665 nm (UV-vis). The UV radiation source was 365 nm cross-linking oven (DYMAX ECE 5000) (Dymax, Torrington, CT, USA).

## 3. Results and Discussion

### 3.1. Fiber Morphology

**Figure 2:**
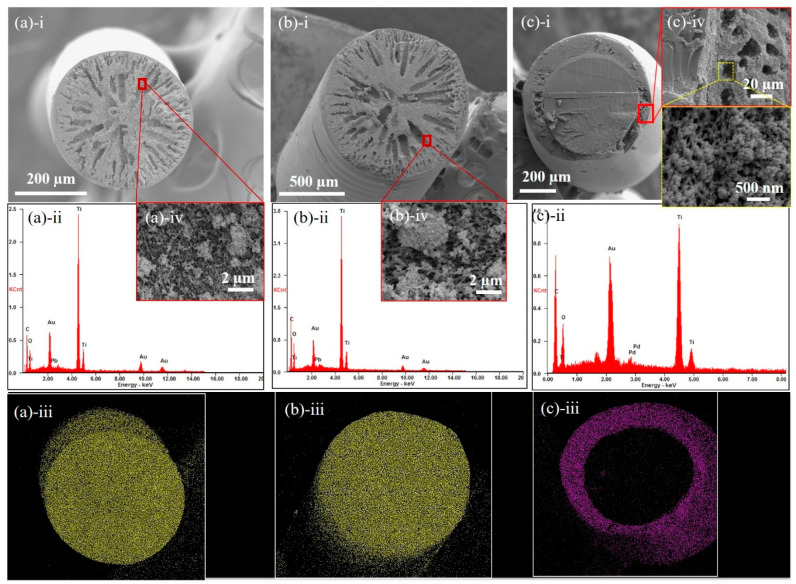
Morphological and compositional characterization of (**a**) D5, (**b**) D10, and (**c**) M-phase fibers with (**a-i** to **c-i**) the cross-sectional surface topology, (**a-ii** to **c-ii**) EDS of the surface showing the element identification, (**a-iii** to **c-iii**) distribution of TiO_2_, and (**a-iv** to **c-iv**) zoom-in regions in (**a-ii** to **c-i**) showing the dispersion of TiO_2_.

The fiber morphology influences both the mechanical durability and photocatalytic behaviors. During our fiber-making process, the choices of solvent (i.e., DMAc) and nonsolvent (i.e., DI water) were critical in forming the desirable microstructures (Figure 1a). PMMA is a suitable substrate material, since it is ~80% transparent to radiation with a wavelength of 250 nm or above. However, the introduction of pores in the substrate hampers its optical properties. A porous substrate demonstrates poor light transmittance through its thickness since it scatters most of the light falling on the surface (Figure S1). Our TiO_2_ sample had a bandgap of 3 eV (or λ = 413 nm, Figure 1b), which means that the substrate should have been transparent to radiation with a wavelength of less than or equal to 413 nm. TiO_2_ only anchored to the surface of the porous fibers that had access to light essential for photocatalysis. This corroborates the need for lean fiber-like architectures whose three-dimensional (3D) structure provides a greater avenue for light impingement. The overall structure of the D-phase was porous, with uniformly dispersed TiO_2_ (Figure 2a,b). PMMA filaments extruded at a temperature of 275 °C (i.e., higher than the glass transition from Differential Scanning Calorimetry in Figure 1d) had 0.5 and 1.0 mm diameters, respectively (i.e., D_5_ and D_10_), based on the extruder sizes. The D_5_ fiber had a ~two times higher specific surface area (SSA) compared to D_10_ (Table 1). This was because of the different sizes of the fiber samples, which influenced the coagulation dynamics [24]. The solvent leaching process was more effective for a smaller fiber diameter (D_5_) due to the shorter diffusion scale for the coagulant, resulting in a more porous structure. A consequence of porosity was mechanical fragility, as can be seen in the D-phase fibers (Table 1). As a comparison, the M-phase fibers with a densely-packed core region surrounded by a porous sheath (Figure 2c) and uniform TiO_2_ dispersions exhibited a high mechanical robustness (Table 1). Note that, as shown in Figure 2a-iii to Figure 2c-iii, the TiO_2_ was homogeneously dispersed in both the dispersed and core-shell fibers, which is critical for studying the microstructural effects on their water treatment efficiency. The TiO_2_ concentration in both the D-phase and M-phase surface was 36 wt.% (Figure 1e). This concentration of TiO_2_ was selected due to the fact that higher TiO_2_ concentrations in DMAc will cause particle aggregation and fast sedimentation that will influence the coating quality on PMMA fibers. A densely packed core was desirable, since it implies that the core has no visible pores or voids, which might hamper light transmission.

Methylene blue is frequently used to dye office supplies and color papers, as well as to tone up silk colors. In veterinary and human medicine, MB has been largely used in several therapeutic and diagnostic procedures. Natural degradation through conventional water treatment procedures is challenging because of MB’s resistance to light, temperature, water, and chemicals. The long-term existence of MB in nature can not only pollute the water system, but also pose dangers to human health. Photocatalytic oxidation is considered to be one of the most effective ways of degrading MB.

**Figure 3:**
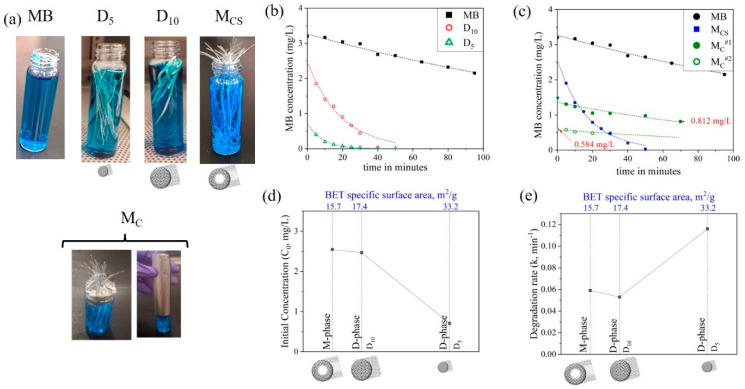
(**a**) Samples used for photocatalytic testing. Photocatalytic removal of methylene blue (MB) in the presence of UV light in the (**b**) D-phase, and (**c**) M-phase fibers. Degradation parameters (**d**) C_0_ and (**e**) k estimated using regression fit as a function of the BET specific surface area (SSA).

Four sets of fiber samples (D_5_, D_10_, M_C_, and M_CS_), each containing ~50 mg of TiO_2_, were placed in individual glass vials containing 8 mL MB solution (3.2 mg/L) (Figure 3a). The glass vials were kept in the dark (i.e., inside a closed opaque box to protect them from light exposure) for five days to achieve the adsorption-desorption equilibrium of MB. Then, the samples were exposed to UV radiation and the MB dye concentrations were monitored over time (Figure 3b,c). The photocatalytic reaction kinetics was analyzed using the Langmuir–Hinshelwood pseudo-first-order kinetics model [25].

(1) $$\ln\left(\frac{C}{C_{O}}\right)=-k_{MB}t$$

Here, *C*_0_ and *C* represent the dye concentration after the five-day soaking period and during UV exposure, respectively. *k*_MB_ represents the degradation rate and time, *t*, was recorded from the instant when the sample was exposed to UV radiation. The parameters $C_{0}$ and k were estimated by regression fit (Table 1). The experiments were categorized into two sets. First, to analyze the overall photocatalytic performance of D-phase (D_5_ and D_10_) and M-phase (M_CS_). Second, to quantify the effectiveness of the fiber core in M-phase fiber (M_C_). The sample was covered in aluminum foil to only allow light transmission through the core (Figure 3). An MB solution without any fibers was used as a control test.

### 3.2. Overall Photocatalytic Performance

The MB concentrations (*c*) as a function of time (*t*) for D_5_, D_10_, and M_CS_ fibers exposed in MB solutions are shown in Figure 3b,c. The y-intercept depicts the initial MB concentration C_0_, which is characteristic of the adsorption capability of the fiber. D_5_ fibers demonstrated the lowest C_0_, corresponding to the highest porosity (Table 1) for contaminant adsorption. On the contrary, D_10_ and M_CS_ had a comparable BET SSA, resulting in comparable C_0_ (Figure 3d). The decrease in MB concentrations over time (*y*-axis in Figure 3b,c) was a result of the photo-inactivation of adsorbed contaminants. UV radiation decomposed the adsorbed dye, which facilitated the adsorption of fresh MB from the solution into the replenished sites in the fiber. Therefore, the degradation rate (column *k*_MB_, Table 1) was a measure of the substrate’s morphological ability to deliver UV light to the immobilized TiO_2_. As discussed earlier, the good dispersion in both the dispersed and core-shell fibers (Figure 2a-iii to Figure 2c-iii) excluded the influence of TiO_2_ agglomerations, and thus, the differences in the degradation rate and initial concentrations were the results of the fiber microstructures. Amongst all of the samples, D_5_ demonstrated the highest degradation rate of 0.116 min^−1^. This was attributed to the lean architecture of D_5_, which ensured better light delivery. M_CS_ and D_10_ exhibited comparable degradation rates, in spite of M_CS_ being comprised of a fiber core, which entailed better light accessibility. It should be noted that our test of the degradation of water pollutants was based on a simple setup with UV light radiating toward a bundle of randomly oriented fibers with both cross-section and surfaces exposed. This setup eases the practical application; however, precise UV transition toward exposed fiber ends may reveal the role of the core in pollutant degradation, especially for M_CS_ fibers when the core is expected to transmit UV-light.

### 3.3. Effectiveness of the M-Phase Core

M-phase fibers with only fiber cross-section areas exposed in MB solutions were used to check the UV-transmittance efficiency of the core (M_C_^#^). For M_C_^#1^, the MB concentration reduced during UV exposure, indicating that the fiber core was successful in delivering light in the M-phase architecture (Figure 3c); however, this degradation rate, compared to that facilitated by the surface pores, was one order of magnitude smaller (Table 1). Then, the same sample was left in a dark room for another day and retested in the presence of UV (represented by M_C_^#2^). MB concentrations after the first cycle of exposure (M_C_^#1^) and prior to the second cycle of exposure (M_C_^#2^) differed by 0.226 mg/L, implying continued dye adsorption. During M_C_^#2^, the MB concentration reduced again and eventually saturated in ~50 min. Dye decomposition during M_C_^#1^ and M_C_^#2^ indicated that UV exposure facilitated dye adsorption by purging the TiO_2_ sites from the previously adsorbed dye, producing similar degradation rates (i.e., 0.0077 vs. 0.0069, shown in Table 1). The saturation of M_C_^#1^ and M_C_^#2^ curves indicates the asymptotic nature of adsorption, which slows down due to a reduction of concentration gradients. Therefore, the pace of adsorption limits the photocatalytic performance of the fibers.

## 4. Conclusions

In this study, we developed novel porous PMMA-TiO_2_ fibers using wet phase inversion and analyzed their photocatalytic performance. Two fixed-bed architectures were explored, namely, a dispersed phase PMMA-TiO_2_ fiber (D-phase) and multiphase fiber (M-phase) with a solid PMMA core and dispersed PMMA-TiO_2_ sheath. D-phase exhibited the highest degradation rate of 0.116 min^−1^ due to its high porosity of 33.2 m^2^/g. Size-controlled D-phase and M-phase samples with a comparable porosity displayed similar performance characteristics, despite operating on different mechanisms of light exposure. This means that the overall rate was limited by adsorption of the support structure, even though the overall photocatalytic process required concurrent adsorption and photoactivation. From the perspective of product development, internally illuminated M-phase fibers will result in a compact reactor design, since the fibers can be bundled together, whereas the light distribution in externally illuminated D-phase becomes a limiting factor. The influences of a porous morphology and solid core on light transport characteristics were also explored in this study. To further improve the performance of porous fixed-bed reactors, the dimensions and adsorption sites of the fiber must be optimized to balance both mass transport and light accessibility.

## Figures and Tables

**Table 1:** Physical properties of the as-fabricated fibers.

Fiber Morphologies	Sample	Dimensions (*d*, Core Diameter; *t*, Sheath Thickness; mm)	Mechanical Properties ^§^		BET Specific Surface Area (m^2^/g)	Langmuir–Hinshelwood Pseudo-First-Order Kinetics Parameters	
			Young’s Modulus (GPa)	Tensile Strength (MPa)		Initial Concentration *C*_0_, mg/L	Degradation Rate *k*_MB_, min^−1^
D-phase	D_5_	*d* = 0.5	Fragile		33.2	0.706	0.116
	D_10_	*d* = 1.0			17.4	2.466	0.053
M-phase core-shell	M_CS_	*d* = 0.5, *t* = 0.1	7.52 ± 2.73	117.7 ± 43.2	15.7	2.545	0.059
	M_C_^#1^					1.364	0.0077
	M_C_^#2^					0.581	0.0069

^§^ M-phase core-shell and M-phase core showed comparable mechanical properties. Note that due to the porous feature of the M-phase core-shell, it is challenging to precisely estimate the cross-section area. See BET data in Figure S2. ^#1,2^ are core-shell fibers used for the second-cycle measurement of photocatalysis.

## Supplementary Materials

The following are available online at https://www.mdpi.com/2079-4991/10/7/1279/s1, Figure S1. Schematic sketch of light interaction in the (a) S-polymethyl methacrylate (PMMA)_film_, (b) P-PMMA_film_, and (c) Ti-PMMA_film_. Optical properties characterized using UV-vis (d) absorbance (e) transmittance and (f) reflectance. Figure S2. (a) N_2_ adsorption-desorption curve and (b) Barrett-Joyner-Halenda (BJH) pore size distribution for D_5_. Figure S3. (a) N_2_ adsorption-desorption curve and (b) BJH pore size distribution for D10. Figure S4. (a) N_2_ adsorption-desorption curve and (b) BJH pore size distribution for M-phase.
