# Supplementary material for: PMMA-TiO2 Fibers for the Photocatalytic Degradation of Water Pollutants
Source: Nanomaterials (Basel). 2020 Jun 30;10(7):1279. doi: 10.3390/nano10071279 (PMC7407631; doi:10.3390/nano10071279)
Supplement: Supplementary file 1 [file nanomaterials-10-01279-s001.pdf]

nanomaterials

Supplementary Materials

PMMA-TiO_2 Fibers for the Photocatalytic Degradation of Water Pollutants

Namrata Kanth [1], Weiheng Xu [2], Umesh Prasad [2], Dharneedar Ravichandran [2], Arunachala Mada Kannan [3] and Kenan Song [3,*]

1 Materials Science & Engineering, School for Engineering of Matter, Transport and Energy (SEMTE), Ira A. Fulton Schools of Engineering, Arizona State University, Tempe, AZ 85281, USA; nkanth@asu.edu
2 System Engineering, The Polytechnic School (TPS), Ira A. Fulton Schools of Engineering, Arizona State University, Mesa, AZ 85212, USA; weihengx@asu.edu (W.X.); uprasad1@mainex1.asu.edu (U.P.); dravich2@asu.edu (D.R.)
3 The Polytechnic School, School for Engineering of Matter, Transport, and Energy (SEMTE), Ira A. Fulton Schools of Engineering, Arizona State University, Mesa, AZ 85212, USA; amk@asu.edu
* Corresponding Author: kenan.song@asu.edu; +1-(480)-727-2720

Received: 9 June 2020; Accepted: 21 June 2020; Published: date

S1. Optical Properties of a PMMA Matrix with/without Pores

Pores are capable of scattering light, and they can interfere with light transport essential for photocatalysis. To systematically understand the influence of pores in the substrate, the optical properties of porous polymethyl methacrylate (PMMA) with anchored TiO_2 (TiO_2-$PMMA_{film}$) were analyzed. These results were compared to the optical properties of solid PMMA (S-$PMMA_{film}$) and porous PMMA (P-$PMMA_{film}$) substrates. Due to sample size limitations for the UV-vis setup, thin films were fabricated as representative samples. Even though the complexities in the fabrication of films differ with fibers, the findings are qualitatively applicable. $PMMA_{film}$ was fabricated by drying 25 wt.% PMMA/THF inside a fume hood. P-$PMMA_{film}$ and TiO_2-$PMMA_{film}$ were prepared by smearing 25 wt.% PMMA/DMAc and D-phase solution (Figure S1), respectively, on a glass slide, and then placing the glass slide in a DI water coagulation bath to render the structure porous. The UV-transmittance/reflectance mechanism is explained in Figure S1a-c and the UV-vis results are shown in Figure S1d–f. The absorbance and transmittance graphs were directly obtained from the UV-vis. The reflectance curve was plotted using the following equation:

$$R = 1 - A - T. \quad (1)$$

Here, R, A, and T are the fraction of the radiation reflected, absorbed (α/α_{max}), and transmitted, respectively. The S-$PMMA_{film}$ was observed to be transparent to radiation with a wavelength of 250 nm and above. Since S-$PMMA_{film}$ shows no obvious indication of pores, the optical properties are intrinsic to the material. For wavelengths above 250 nm, this film was ~80% transparent, making it a suitable candidate for anchoring TiO_2 nanoparticles. P-$PMMA_{film}$ exhibited less than 1% transmittance, which demonstrates the impact of pores on the substrate. The reflectance of P-$PMMA_{film}$ was ~50% for radiation with a wavelength above 250 nm. Therefore, P-$PMMA_{film}$ shows poor transmittance through the thickness of the film, as it scatters ~50% of the radiation falling on its surface. Evidently, if TiO_2 were anchored on the porous surface, then the scattered radiation would be available for absorption. The optical properties of TiO_2-$PMMA_{film}$ indicate the same. The reflectance curve of Ti-$PMMA_{film}$ is shifted compared to the reflectance curve of P-$PMMA_{film}$. This means that the anchored TiO_2 indeed absorbs radiation scattered by the porous surface. This corroborates the need for lean fiber-like architectures whose three-dimensional (3D) structure provides a greater avenue for light impingement. Pores are capable of scattering light, and they can interfere with light transport essential for photocatalysis.

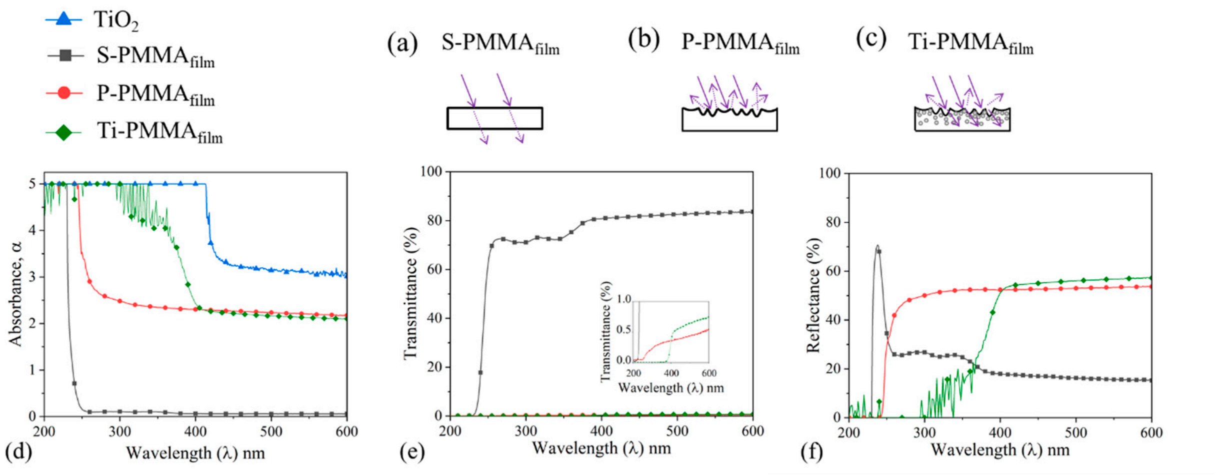

Figure S1. Schematic sketch of light interaction in the (**a**) S-polymethyl methacrylate $(PMMA)_{film}$, (**b**) $P\text{-}PMMA_{film}$, and (**c**) $Ti\text{-}PMMA_{film}$. Optical properties characterized using UV-vis (**d**) absorbance (**e**) transmittance and (**f**) reflectance.

S2. Brunauer, Emmett, and Teller (BET) Porosity Analysis of the Fiber Samples

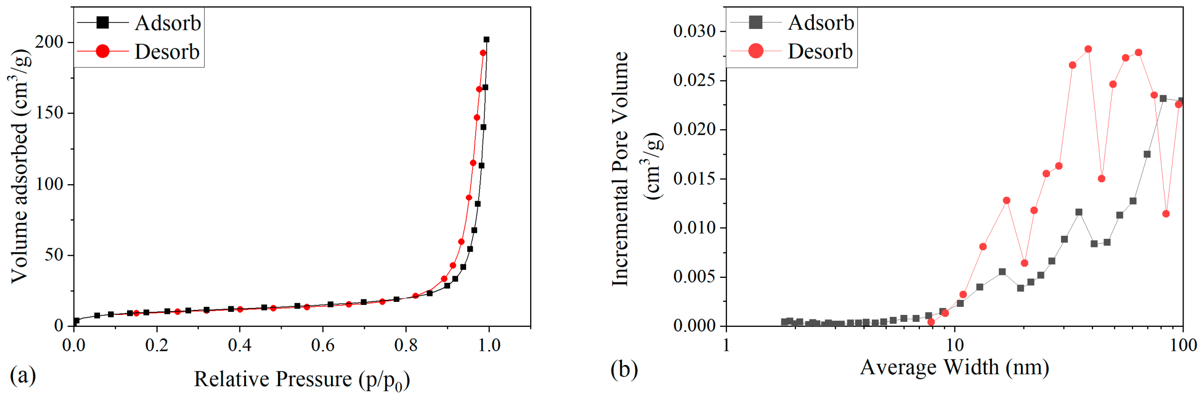

Figure S2. (**a**) N_2 adsorption-desorption curve and (**b**) Barrett-Joyner-Halenda (BJH) pore size distribution for D_5.

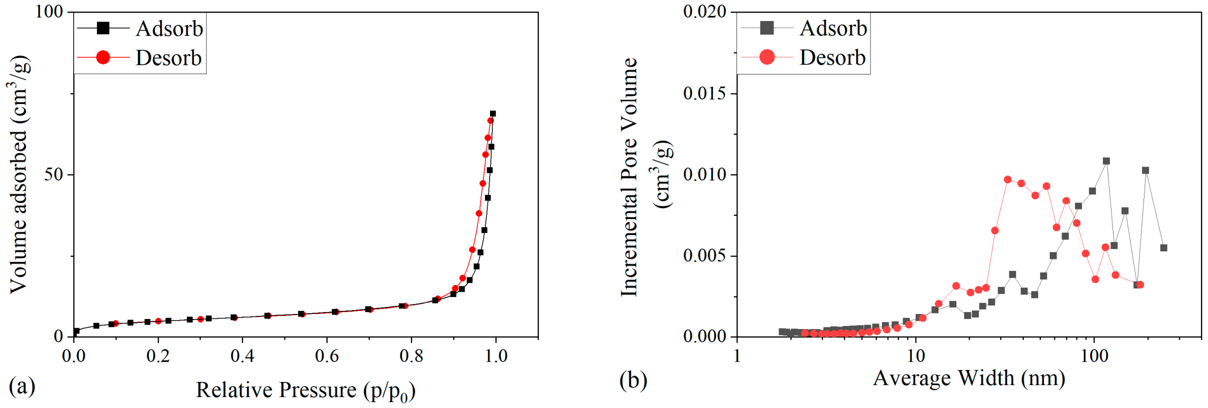

Figure S3. (**a**) N_2 adsorption-desorption curve and (**b**) BJH pore size distribution for D_{10}.

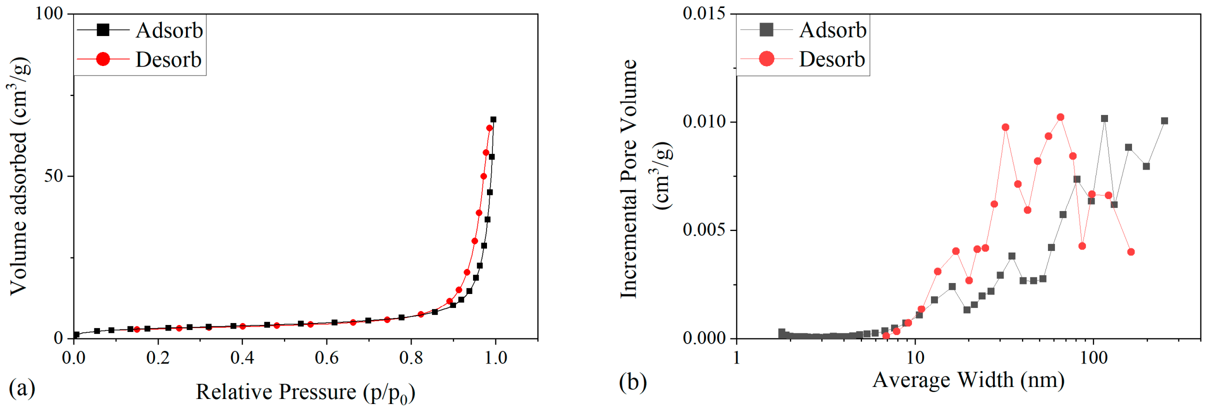

Figure S4. (**a**) N_2 adsorption-desorption curve and (**b**) BJH pore size distribution for M-phase.

The M-phase BET specific surface area (SSA) was scaled to exclude the weight of the non-porous fiber core. Experimentally, the M-phase BET SSA was 11.4229 m^2/g. The weight of the sample was corrected from 0.22 to 0.16 g, resulting in a BET SSA of 15.71 m^2/g.
